# A qualitative exploration of the misconceptions, knowledge gaps and constructs of leptospirosis among rural and urban communities in Malaysia

**DOI:** 10.1371/journal.pone.0200871

**Published:** 2018-07-18

**Authors:** Surianti Sukeri, Zawaha Idris, Wan Mohd Zahiruddin, Mohd Nazri Shafei, Norazlin Idris, Rukman Awang Hamat, Tengku Zetty Tengku Jamaluddin, Malina Osman, Zainudin Abdul Wahab, Aziah Daud

**Affiliations:** 1 Department of Community Medicine, School of Medical Sciences, Universiti Sains Malaysia, Kubang Kerian, Kelantan, Malaysia; 2 Health Promotion Unit, Penang State Health Department, Bangunan Persekutuan, Jalan Anson, Penang, Malaysia; 3 Department of Medical Microbiology and Parasitology, Faculty of Medicine, Universiti Putra Malaysia, Serdang, Selangor, Malaysia; 4 Health Department of Federal Territory Kuala Lumpur and Putrajaya, Jalan Cenderasari, Kuala Lumpur, Malaysia; Imperial College London, UNITED KINGDOM

## Abstract

This qualitative study aimed to explore the misconceptions, knowledge gaps and constructs of leptospirosis among 72 respondents from rural and urban districts in two states of Malaysia. We conducted focus group discussions and data were examined using thematic analyses. The layman term of ‘rat urine disease’ contributed the most to the misconceptions regarding leptospirosis. There were gaps in the knowledge among urban and rural respondents in the two states, with the majority of subjects demonstrating a poor understanding of the disease. Construction of knowledge about leptospirosis relied mostly on the information provided by mass and social media; reading materials; word-of-mouth publicity; observations; experiences; and knowledge sharing among families, friends, and communities. The study findings may provide the foundation for the development of educational materials that may reduce the gaps in knowledge, and thereby improve health literacy and enhance preventive health behaviours for avoiding leptospirosis.

## Introduction

Leptospirosis is a bacterial zoonotic disease transmitted from vertebrate animals to man. It is most commonly observed in the tropical or subtropical countries and is prevalent in both, urban and rural areas. The incidence of leptospirosis ranges from 0.1–1 per 100,000 in temperate climates to 10–100 per 100,000 in the humid tropical regions. The incidence may exceed 100 per 100,000 in the high-exposure risk groups and during outbreaks [1]. Leptospirosis is an emerging public health concern in Malaysia with an incidence rate of 6.99 per 100,000 in 2010 that increased to 30.2 per 100,000 in 2015 [2]. The most notable outbreak occurred during the Eco-Challenge-Borneo Sabah, Malaysia that caused a considerable upheaval because 80 of the 189 athletes from 26 countries were affected [3]. In 2010, four individuals died from melioidosis and leptospirosis co-infection during a search-and-rescue mission at the Lubuk Yu recreational park in Malaysia [4]. The number of deaths from leptospirosis continues to grow with no visible signs of reduction.

Leptospirosis affects farmers, outdoor workers, military personnel, medical personnel, municipal workers, recreationalists, and the general public, thus posing as a vital financial and public health issue [5]. Knowledge regarding the signs, symptoms, and complications of leptospirosis among the public is crucial for early diagnosis, prompt treatment, and proper case management for reducing leptospirosis-related mortality. However, previous studies have shown a lack of such knowledge. For example, a knowledge, attitude, and practice (KAP) survey regarding leptospirosis among 800 households in Trinidad reported that only 52.4% individuals had heard of leptospirosis and approximately 50% had no knowledge about the signs or symptoms of the disease [6]. Moreover, a similar KAP study regarding leptospirosis on 106 municipal workers in Tiruchirapalli, Tamilnadu, found that majority of the workers had poor knowledge (87.2%) regarding the disease [7]. In their study on 460 adolescents in a highly endemic area in Sri Lanka, Samarakoon and Gunawardena found that even though the respondents’ overall level of knowledge regarding leptospirosis was satisfactory, only 52% of them had a good level of knowledge of leptospirosis [8]. A more recent study among 444 rural participants in a rural state in Malaysia revealed 57.0% had poor knowledge level of leptospirosis [9].

However, these findings can be difficult to interpret because of the lack of contextual understanding. It does not explain why some respondents have good knowledge about leptospirosis while others do not. Although these surveys provide information regarding the public knowledge of leptospirosis, to the authors’ knowledge, the gaps in knowledge as well as the various aspects of knowledge construct and misconceptions about this disease are under-researched.

We believe that a qualitative investigation is warranted. Apart from confirming the reported findings, such research would enable the understanding of the construction of knowledge regarding leptospirosis and help determine whether any gaps and misconceptions about leptospirosis exist. To the authors’ knowledge, no other study with this primary focus has been conducted. With this background, the current study was planned to conduct a detailed examination of the public knowledge and perceived severity of leptospirosis; this, according to Vinetz et al., is the first step towards the development of community participatory approaches aimed at reducing the incidence of leptospirosis [10]. The findings of the present study may assist in the improvement and development of an appropriate leptospirosis educational program and supplementary materials.

## Materials and methods

### Settings

The study was carried out in Kelantan and Selangor, the two states that recorded the highest cases of leptospirosis in Malaysia [11]. Kelantan has an area coverage of 15105 km^2^ and a population of 1.8 million, while Selangor has an area coverage of 7930 km^2^ and a population of 6.3 million. The two states differ in terms of their economic status, with the gross domestic product (GDP) growth of Selangor being 5.7% and that of Kelantan being 3.5% [12]. Rural areas are defined as gazetted areas with a minimum population of 10,000 where 60% of the population (aged ≥ 15 years) is involved in non-agricultural activities. Those areas that did not fulfil these criteria were considered urban areas [13].

### Research team

Four members comprised the research team; these researchers had a combined experience of 20 years in qualitative research; ZI, NJ, and ABR were experienced researchers from the Institute of Behavioural Research, and SS was a senior university lecturer. All the members held postgraduate degrees and had undergone qualitative method trainings in Australia and Germany. They worked in close collaboration to construct the interview schedule; recruit participants; and collect, analyse, and interpret the data. No team member had a prior relationship with any study participant.

### Study design

The study used the phenomenology method. We conducted focus group discussions (FGD) because of the group dynamics that encourages the subjects to respond or behave in unanticipated ways, evoke information that relates to the emotional processes and inner reasons, thus providing more opportunity for intuitive research [14].

### Study sample

The number of focus groups depends on the complexity of the research question and the composition of the groups; however, as per the rule of thumb, there should be between two and five groups per category. However, the inclusion of few more than the suggested number of groups is recommended to account for any unforeseen confounders [15]. We decided to form four groups for each category. To ensure group synergy and homogeneity, participants were grouped according to their respective rural/urban categories. Considering the explorative nature of the study, the recruitment of respondents was based on the sole inclusion criterion of being fluent in the Malay language i.e. the language used during the interview. Participants were purposively selected from rural and urban districts of Kelantan and Selangor. Participants from the rural districts in both states were recruited by the community leaders, while urban participants were enlisted among those who responded to the open invitations circulated via the university email of the research team.

### Data collection

The study was conducted between November 2015 and December 2015. The FGD sessions were conducted at the research institution and at the houses of the community leaders. During all sessions, a physician who could answer any medical queries of the participants was present. The interviewer (ZI) was a research fellow from a non-clinical background. ZI professional background, knowledge, experiences and prior assumptions neither interfere with the study direction nor influence participants’ willingness to talk openly.

The number of participants for each group was limited to eight to ten, and each session of each group was facilitated by two moderators. The research team drafted an interview guide that comprised questions regarding awareness, risk factors, mode of transmission, signs and symptoms and prevention of leptospirosis. Participants were also asked what influenced their current understanding and perspectives about the potentially useful educational tools.

The FGDs were audio-recorded and ranged in duration from 45 to 80 minutes. The transcription of the interviews was prepared in the Malay language. Field notes and reflections on the interviews were maintained and included in the data analyses. Data saturation was achieved after no new information emerged, and data collection ceased after the eighth FGD. All participants were offered MYR50 cash in recognition of their contribution to the study.

This study was conducted in strict accordance with the Declaration of Helsinki. The National Medical Research Registry and the Human Research Ethics Committee of the Universiti Sains Malaysia (USM/JEPeM/15120552) approved the study protocol. Informed written consent was obtained from all the study participants. We adhered to the COREQ checklist while reporting the study findings.

### Data analysis

SS and ZI performed the data analyses. For this study, thematic analysis was used for identifying, analysing, and reporting the patterns within the data. The steps used to create the meaningful themes included familiarisation with the data, generation of the initial codes, identification of the themes among the codes, review of the themes, definition and naming of the themes, and production of the final report [16]. In the first step, each transcript was read and reread, and the initial ideas or impressions were recorded. This step was performed simultaneously with the on-going interviews and helped the development of the overall idea. The second step involved the production of the initial codes from the data. Sections of the text were organized into codes using NVivo 11 (QSR International, Melbourne, Australia) based on the categories covered in the interview guide, and inductively as the text was digested. Concurrently and inductively, the codes were organized into broader categories. The deviant cases were investigated in depth to explore the reasons for the contradictory or unusual views that were expressed. To minimize interpretation error, all analysis were performed in Malay language. Relevant quotes were translated word-for-word from Malay to English language and were checked by a professional.

## Results

There were eight FGD sessions involving 72 participants; two sessions were held at each of the selected two rural and urban locations in Selangor and Kelantan. Both, the rural and urban respondent groups comprised nearly the same number of subjects (38 vs. 34, respectively). None of the respondents had history of leptospirosis infection. The age of the study participants ranged from 18 to 79 years. Majority of the urban participants were diploma/degree holders and had desk jobs, while most subjects from rural areas were self-employed, retirees and housewives who received high school education.

The study findings are summarised in Table 1. A detailed explanation of the findings is described in the following subheadings.

**Table 1:** Summary of the study findings.

Main findings	Description of the main findings
Misconceptions regarding leptospirosis	Misconception regarding leptospirosis that stemmed from the usage of the layman term of ‘rat urine disease’
	Rats are the sole vectors and anything related to rats causes leptospirosis
	Rural rats are harmless and that immigrant workers were more susceptible for infection
	Leptospirosis is a viral disease
	Availability of an ‘antivaccine’
	Similarity of the treatment with that of dengue
Gaps in the knowledge regarding leptospirosis	Unfamiliar with the term leptospirosis
	Had poor to basic knowledge regarding leptospirosis source of infection, its route of transmission, risk factors and preventive measures
Construction of knowledge	Construction of knowledge was mainly based on the layman term
	Developed through reading, own observations and analyses, experience and history of disease, discussions between friends and families
	Poor knowledge construct due to the lack of health campaigns by the health officials
	Knowledge attained from information disseminated via the television, newspapers, social media, and word-of-mouth publicity

### Misconceptions regarding leptospirosis

There was a misconception regarding leptospirosis among both, rural and urban participants that stemmed from the preferred usage of the layman term. The usage of the layman term led to the common belief that rats are the sole vectors and anything related to rats causes leptospirosis,

“Leptospirosis is rat urine disease. It is transmitted through rat faeces, rat urine, or whatever the rat touches.” (urban)

The most common response given for the question regarding the route of transmission was that it is transmitted through the ingestion of infected drinking or recreational waters as well as food and kitchen utensils that have been contaminated by rat urine and rat droppings,

“Leptospirosis can occur if there is rat urine in the kitchen and it comes into contact with the spoons, plates, or the refrigerator.” (rural)

It was also believed that rats in the market areas posed a higher risk because rural rats are ‘cleaner’,

“The disease is only present in the marketplace. I have rats in my house that urinate everywhere; however, it's been three years, and I'm still fine, which means that the urine of house rats has no germs and is not dangerous.” (rural)

They also believed that only immigrant workers were more susceptible,

“I believe that the immigrants are more prone to leptospirosis infection because they eat and simply throw away their food waste; this can lead to rat infestation.” (urban)

Some believed that leptospirosis is a viral disease,

“This is a new virus; so, no antiviral has yet been discovered.” (urban)

while others thought that there is an ‘antivaccine’,

“I believed there is an antivaccine for leptospirosis.” (urban)

There was also a misconception about the treatment. Few respondents confused its treatment with that of dengue,

“I don’t know the treatment process, but logically, when germs enter the body, blood needs to be detoxified to eliminate these germs from the body, maybe by administering a saline drip or consuming a glucose drink.” (urban)

### Gaps in the knowledge regarding leptospirosis

We observed that only few respondents exhibited a good level of knowledge about leptospirosis; further, majority of them were from the urban areas in both states. These groups of respondents were able to provide detailed and accurate descriptions of the causes, risk factors, route of transmission, symptoms, and complications such as severe clinical manifestations in the form of jaundice, acute kidney injury, and pulmonary haemorrhage syndrome.

Meanwhile those with basic knowledge in both, the rural and urban areas could partially identify the causes of the disease, its route of transmission, symptoms, and prevention. They were also aware that untreated leptospirosis could lead to death and that they should seek emergency treatment if they suspected that they were exposed to it.

However, the remaining the participants in both settings had poor knowledge regarding leptospirosis. These groups of respondents initially acknowledged that they did not know what leptospirosis was and were unable to attempt any explanation on direct questioning. However, when the layman term ‘rat urine disease’ was introduced, some knowledge and understanding was often revealed,

“Leptospirosis is probably a western term. If you ask me, I panic whenever I hear it. But if you say rat urine disease, then I do not panic because I know this term. With respect to rural people, even though they might have heard of it, they would not know much about the disease.” (urban)

Many of these participants were also curious about the type of rodents that carry the bacteria,

“I don’t know which species of rat is responsible for this disease. There are many rats here in the village, is it those rats or some other types of rats?” (rural)

Majority of the respondents were unsure of the disease complications and did not know that leptospirosis can be transmitted through direct contact with the urine or tissue of an infected animal through the skin mucus membrane or through indirect contact of broken skin with infected soil and inhalation of droplets of infected urine. The persistence of the *Leptospira* bacteria that can remain viable in soil and stagnant water was not mentioned. The study participants were also less informed about the risk factors for leptospirosis. Despite Kelantan being a state at high-risk of floods, 50% of the responses did not associate leptospirosis with flood; further, majority did not know that leptospirosis is an occupation-related disease and that being barefoot presents a high risk.

Even though most of the respondents could give accurate descriptions of the disease signs and symptoms, none was aware that an individual can only be a carrier without any symptoms and that the complications range from atypical fever to death. Majority of the respondents also recognized the necessary preventive and control measures; however, the significance of prophylaxis and the vaccination for animals was not discussed, suggesting lack of knowledge regarding this subject. The use of personal protective equipment was only mentioned by two participants

### Construction of knowledge

In this section, we present the findings of our attempts to understand how knowledge was acquired and understood. It appeared that for many respondents, the understanding of the causes of leptospirosis was based on the layman term,

“I may not know what leptospirosis is, but if it is called rat urine disease, then it means that the disease is caused by rats, right? Is the disease not caused by chickens, cats or cows?” (urban)

The construction of knowledge was also developed through reading and discussions with friends,

“What I understand from other people’s discussion about leptospirosis is that it is rat urine disease. Further, from what I have read in articles, it is contracted when one swims in river, accidentally swallows water from the river, or drinks canned beverages that have been contaminated by rats without washing it first. Rats may have run over the top of the cans and peed on it.” (urban)

Few respondents were aware of the disease owing to the information disseminated via the television, while others obtained the information from newspapers,

“I have never heard of it before. Within the last 2–3 years, a lot of people have been infected by this rat urine disease, and some have even died from it. Before this, I knew nothing about this disease. What I now know is mostly from the information seen on the TV and the newspaper.” (rural)

Knowledge does not necessarily need to be taught; an individual can arrive at an accurate conclusion based on his/her own observations and analyses,

“I met this patient in the ward who was first treated for dengue; however, a subsequent blood test confirmed it to be leptospirosis. This means this disease has symptoms similar to those of dengue.” (urban)

Knowledge is also reinforced through experience and knowledge sharing among family members

“I know about the disease because I know someone who had it. My friend died from leptospirosis two years ago” (urban)

“I have a family member who works at the hospitals who tells me about patients and their conditions.” (rural)

Conversely, poor understanding on leptospirosis occurred because it was not discussed within the local communities.

“I don't know much about this disease as I have never heard of it before. There was no case of leptospirosis in the village unlike dengue that was very common.” (rural)

Respondents also commented that they had poor knowledge regarding leptospirosis because they had never been affected by this disease.

“There should be a health education program for the prevention of this disease. Otherwise, we wouldn’t know anything about it unless a family member is infected.” (urban).

We discovered that the understanding about leptospirosis also stemmed from the lack of health campaigns on the disease,

“Health campaigns on leptospirosis are rarely seen; maybe it is a new disease. There is no campaign on the prevention of leptospirosis. All the roadside banners or billboards only talk about dengue.” (urban).

We also asked respondents about their preferred mode of health education for leptospirosis. The common responses were mass media, talks and exhibitions in health clinics and schools, Friday prayer sermons, pamphlets, posters, banners, and campaigns. Respondents from the rural areas in Kelantan preferred more traditional methods of education dissemination within the vicinity of their villages and local surroundings, such as get-together clean-up activities, demonstrations, talks, and movie showings in the village. Younger, internet-savvy respondents named the social media as their preferred platform.

## Discussion

This qualitative study aimed to explore the gaps in knowledge, misconceptions, as well as the context of knowledge construction about leptospirosis among respondents in rural and urban areas of Kelantan and Selangor. This study demonstrated that almost all the participants had heard of leptospirosis or the layman term used for it: rat urine disease. However, when probed, majority of the respondents were found to have poor to basic knowledge regarding leptospirosis. This finding corroborates with previous KAP studies conducted elsewhere, including Selangor and Kelantan that have reported that even though 52.4%–97% of the subjects had heard of leptospirosis [6, 9, 17, 18], 48%–98% had poor knowledge regarding the disease [8, 9, 18, 19].

We discovered that respondents in urban settings and those who were more educated had good knowledge regarding leptospirosis. These findings are in agreement with previous reports that have shown that the knowledge regarding leptospirosis is influenced by the geographical location [6] and education [20] of the respondents. However, it is noteworthy that in this study, respondents from both, rural and urban areas had similarly poor knowledge regarding leptospirosis. According to Stull et al., despite the government initiatives undertaken during the past several decades to increase the awareness regarding zoonotic diseases in the population, public knowledge does not appears to have improved [21]. Our results support this statement. It appears that many respondents in the study still lack the basic knowledge regarding leptospirosis; this is most likely owing to societal complacency. Compared to the more prevalent diseases such as dengue, there are fewer human cases of leptospirosis in Malaysia each year. Therefore, people may be less interested about this deadly disease. A study that evaluated the awareness level regarding zoonosis compared to that regarding trichinosis, rabies, scabies, brucellosis, tuberculosis, and anthrax among rural workers reported that leptospirosis was among the least known zoonosis diseases [22]. The poor knowledge regarding leptospirosis may also be attributed to the low levels of health literacy, an independent predictor of patients’ knowledge [23].

The most notable finding was that the use of the layman term may have formed the basis for the misconception regarding the disease. Leptospirosis is endemic to Malaysia and poses a considerable risk of outbreaks. Thus, if the layman term of the disease that focuses on rodents continues to be used, the challenge in communicating the correct information about the route of transmission for leptospirosis will persist. Misconceptions regarding leptospirosis are common; respondents in India, Malaysia, and Trinidad believed that leptospirosis can be transmitted through mosquito bites [18], may cause lung cancer [7], and can be treated with ‘bush’ medicine [6].

Our survey demonstrates that many respondents had a poor understanding of the detailed aetiology and risk factors of leptospirosis. Living in rural areas, higher exposure to floods and heavy rains, poor living conditions, proximity to sewage, lack of sanitation, and behavioural factors such as walking barefoot and having uncovered wounds are proven risk factors of leptospirosis [24], all of which were rarely mentioned by our respondents. Our findings contrasted with those of studies on the communities within the parish of St. Mary, Jamaica that had a high level of awareness about the health risks associated with flooding [17]. Moreover, majority of our study respondents did not cite the use of personal protective equipment such as boots or gloves during gardening or working with livestock as preventive measures against leptospirosis. It is believed that the proportion of households that followed protective practices against zoonotic infections was considerably influenced by the community to which the household belonged [25]. For example, in a study on risky practices related to leptospirosis among a sample of rural school adolescents in Sri Lanka, only 18% reported using gloves and boots 'frequently', while 61% of students who handled cattle or buffalo reported washing their hands and feet after handling the animals [8].

While discussing the patterns of knowledge construction in leptospirosis, we discovered that respondents mostly relied on mass and social media; reading materials; word-of-mouth publicity; observations; experience; and knowledge sharing between families, friends, and communities for information regarding the disease. This was a social process that involved a variety of largely local ‘actor networks’ such as humans as well as paper and electronic media as sources of evidence [26]. In comparison to a local study in Selangor, the main sources of leptospirosis information were the television (49%) and the newspaper (31%) [18]. Findings revealed that information dissemination through the television media was the number one prevention and control strategy to avoid leptospirosis and could be one of the information topics they could share with their family and friends [27]. However, we found that respondents did not mention health care providers as sources of leptospirosis-related knowledge. According to the literature, this may be attributed to the lack of knowledge of zoonosis among healthcare providers themselves [28] because many respondents reportedly felt that medical professionals, particularly physicians and nurses, were themselves not well informed regarding the diagnosis of leptospirosis or the health-education initiatives required to protect people from contracting the infection [10]. This result is further confirmed by the frequent remarks on the lack of health education and promotion programs pertaining to leptospirosis in Malaysia.

It was surprising to note that in this study, the knowledge regarding leptospirosis acquired from social media sites and non-formal channels such as observation, experience, and sharing sessions between friends and families tended to have better retention with the respondents. Although the acquired knowledge may not be completely accurate, respondents could recall the details even though they had learned about it several years previously. Moreover, the knowledge construct of leptospirosis among the elderly who lived in rural areas was greatly dependent on their social circle. The history of leptospirosis among family members also contributed to the formulation on the understanding of the disease. As exhibited by Mohan et al., the chances of a person having knowledge about leptospirosis and its symptoms were higher in areas with higher prevalence rates [6].

We also discovered that respondents who received formal training regarding leptospirosis were able to interpret the disease to a significantly more correct degree. These findings reiterate the importance of health education in improving the knowledge about leptospirosis. Public health officials may consider the recommendations provided by the respondents as a good indication of the acceptance towards health promotion initiatives.

This was a qualitative study; thus, interpretation and generalizability of these findings may be limited. Another limitation is the lack of triangulation. Nevertheless, the results are in agreement with previous findings, and we believe that the absence of triangulation did not reduce the validity of the findings to a great extent. It is possible that some biases were introduced by the moderator effect. However, there were only slight differences between the groups, suggesting minimal bias. Efforts to address stability and consistency the research include group debriefings, multiple researchers, recorded audit trails, and field notes.

## Conclusion

The present study found that the knowledge regarding leptospirosis is poor among the general population. Awareness of a disease is a prerequisite for effective prevention; thus, limited leptospirosis knowledge displayed by the study respondents is concerning. Special attention needs to be given for imparting knowledge among the underserved and high-risk communities in rural settings. There is considerable scope of improvement in the content of the health education material for leptospirosis, with a strong focus on bridging the gaps of knowledge, as highlighted in the study. The usage of the medical term is necessary to effectively address the misconception about the disease that results from the widespread use of the layman term. It was also evident that leptospirosis has remained a neglected disease, and few government initiatives have addressed this public health need for awareness regarding the disease. We believe that our findings provide the rationale for the health authorities to perform a collective professional responsibility and step up health education initiatives that are currently lacking in Malaysia. This investigation also revealed an interesting pattern in the knowledge construct that implied that knowledge dissemination should not be done only via those sources that the government believes should be used by the public, but also via those that are actually used by the public. Health educators need to be aware of their target population’s knowledge levels, health literacy skills, and mode of preference because this may enhance the knowledge gain by individuals, using any of the several routes used for disseminating health education. For instance, imparting education regarding leptospirosis to opinion leaders whom most rural residents approach for gaining knowledge might be a highly effective method. Apart from the mass media, the social media must be recognized as a popular platform for knowledge transfer and used optimally. Leptospirosis prevention in endemic areas is highly dependent on the health education initiatives that enable communities to translate their knowledge into preventive health practices. Community education regarding leptospirosis should thus be considered a priority. The present findings provide useful information that may assist in the planning of future health campaigns and educational materials for leptospirosis.
